# Cardiomeds, an mHealth App for Self-Management to Support Swiss Patients With Heart Failure: 2-Stage Mixed Methods Usability Study

**DOI:** 10.2196/63941

**Published:** 2025-01-15

**Authors:** Lisa Simioni, Elena Tessitore, Hamdi Hagberg, Aurélie Schneider-Paccot, Katherine Blondon, Liliane Gschwind, Philippe Meyer, Frederic Ehrler

**Affiliations:** 1 Faculty of Medicine University of Geneva Geneva Switzerland; 2 Department of Cardiology University Hospital of Geneva Geneva Switzerland; 3 Department of Computer Science University Hospital of Geneva Geneva Switzerland; 4 Medical Directorate University Hospital of Geneva Geneva Switzerland; 5 Department of Pharmacy University Hospital of Geneva Geneva Switzerland

**Keywords:** usability, medication, mobile health, mHealth, Cardiomeds, mobile app, patient empowerment, eHealth, smartphone, heart failure, HF, chronic disease, interactive, self-monitoring, usability test, mobile phone

## Abstract

**Background:**

Mobile health apps have shown promising results in improving self-management of several chronic diseases in patients. We have developed a mobile health app (Cardiomeds) dedicated to patients with heart failure (HF). This app includes an interactive medication list; daily self-monitoring of symptoms, weight, blood pressure, and heart rate; and educational information on HF delivered through various formats.

**Objective:**

This study aimed to perform a mixed methods usability study of Cardiomeds.

**Methods:**

Smartphone users with HF were recruited from the HF outpatient clinic at the University Hospital of Geneva. The usability test was conducted in 2 stages, with modifications made to the app after the first stage to address major usability issues. Each stage required 10 participants to perform 14 tasks, such as entering vital signs, entering a new medication and time of intake, or finding information about HF. Each task was timed, sessions were recorded, and all data were anonymized. After completing the tasks, patients completed the System Usability Scale 10-item questionnaire and answered 5 open questions about their perceptions of Cardiomeds.

**Results:**

Twenty patients with HF, 75% (15/20) of whom were men, with a mean age of 55 years, were included in this study. The average time to complete all 14 tasks was 18 (SD 5.7) minutes. Manual medication entry was the most time-consuming task, taking an average of 154.40 (SD 68.08) seconds in the first stage, 103.10 (SD 42.76) seconds in the second stage, and 128 (SD 63) seconds overall. The mean overall success rate was 77% (SD 0.23%) for the first stage and 94% (SD 0.07%) for the second stage. A total of 30% (3/10) of participants in the first stage completed all tasks without any help compared with 50% (5/10) of participants during the second stage. The average System Usability Scale score was 80% (SD 17%), showing a slight increase from 79% (SD 16%) in the first stage to 80% (SD 28%) in the second stage, which qualifies the app as “good” in terms of usability. Between the 2 stages, part of the app interface was redesigned to address the key issues identified in the first stage. Despite these improvements, problems related to guidance were frequent and comprised 36% (8/22) of the problems in the first stage and 40% (6/15) in the second stage. In response to open questions, 85% (17/20) of the participants responded that they would like to use the app when it became available.

**Conclusions:**

The usability test indicated that Cardiomeds is a suitable and user-friendly app for patients with HF. The app will be further tested in a randomized clinical trial (2022-00731) after acute HF hospitalization to assess its impact on patients’ knowledge about HF, self-care, and quality of life.

## Introduction

### Background

Heart failure (HF) impacts around 2% of the adult population in Europe and is the leading cause of hospital admissions among individuals aged >65 years. Within a year after being hospitalized for acute HF, over half of the patients are readmitted, and nearly a quarter of them die. This situation poses an enormous medical and economic challenge [1]. The cornerstone of HF management is pharmacotherapy, which involves the combination of multiple drug classes [2]. Patients with HF need to make lifestyle adjustments to prevent deterioration, keep track of symptom changes, and take daily medications. Support interventions for self-management behavior generally aim to provide patients with the skills to actively manage their chronic condition. These interventions focus on promoting symptom monitoring and improving problem-solving and decision-making abilities for medical treatment and a healthy lifestyle [3]. According to the latest guidelines of the European Society of Cardiology, self-management strategies are strongly recommended to reduce the risk of hospitalization and mortality related to HF [2]. Various self-management strategies are possible, such as multidisciplinary care, peer meetings, and the use of logs for symptom monitoring or setting goals. The longer these interventions are maintained, the greater the reduction in mortality, with a 1% to 4% decrease in risk for each additional month. In addition, these interventions work better when accompanied by regular contact with health professionals [4].

With advancements in technology, the introduction of therapeutic monitoring with telephone support is becoming increasingly prominent [5], which includes features to register symptoms, enter a treatment card, organize appointments, record alarms for taking medications, or support behavior reinforcement through educational materials [6].

Research consistently supports the effectiveness of structured telephone support and noninvasive telemonitoring. Several systematic reviews have concluded that these approaches significantly reduce all-cause mortality and HF-related hospitalizations [7-9]. Moreover, these interventions also showed enhancements in health-related quality of life, knowledge of HF, and self-care behaviors [7,10].

Smartphone-guided interventions in HF are growing in significance and effectiveness, with studies incorporating monitoring not only through text messages and phone calls but also via mobile health (mHealth) apps [11,12].

Thus, many mHealth apps are being developed and are the subject of various studies [5,6,10,12,13].

Several mHealth apps, providing valuable support to patients with HF are already available. For instance, the WOW ME 2000mg—Heart Failure Self-management Tool (AtlantiCare) focuses on helping patients and caregivers track symptoms and medications [6,14,15]. WebMD (WebMD Health Corp) offers a wide range of health resources, including HF management [14,16]. Similarly, My Cardiac Coach (American Heart Association) is designed for post–heart attack care and self-monitoring. Finally, “Heart Failure Health Storylines” (Heart Failure Society of America) aids in symptom tracking and treatment adherence [6,14,17]. Nevertheless, none of these apps refer to the Swiss medication database.

Experience with these existing mHealth apps has demonstrated a significant improvement in therapeutic adherence among patients requiring daily treatment [18,19]. In addition, patients understood their illness better, which allows them to feel more secure [10].

Despite the potential benefits of mHealth apps, studies have shown that patients with HF often face barriers to achieving good adherence. For many older patients, difficulties begin with technical issues, such as challenges in installing or navigating the apps on their smartphones [20]. These issues are often exacerbated by limited digital literacy, a lack of confidence in using technology, or cognitive impairments commonly associated with HF. In addition, usability concerns, such as complex interfaces, insufficient personalization, or lack of reminders, can further reduce the likelihood of long-term adherence [21]. Overcoming these barriers requires designing apps with simplicity, user-friendliness, and accessibility in mind while providing continuous support for patients [22].

In Switzerland, according to a study conducted by Pro Senectute, 69% of citizens older than 65 years own a smartphone, 81% of whom use it daily. Moreover, this study also discovered that their interest in mHealth apps is consequent [23]. Thus, the familiarity of older adults with smartphones makes the use of an mHealth app credible for conducting a study on patients with HF [24].

In this context, the cardiology department of the University Hospital of Geneva (HUG) has undertaken the development of a new mHealth app tailored for patients with HF, known as Cardiomeds. Designed to complement the physician-patient relationship, the app has been developed following a user-centric approach. We collected the information required to develop the app through interviews and focus groups that included patient groups, clinicians, and IT professionals. We created personas and user journeys and refined our prototype iteratively using feedback from the stakeholders.

Therefore, it was important to conduct a usability test of this app before offering it to patients with HF. Indeed, these tests make it possible to point out the design problems of the apps, to improve the uncovering of opportunities, and to learn about the target user’s behavior and preferences [25].

### Objectives

The study aimed to evaluate the usability of the Cardiomeds mHealth app through qualitative and quantitative usability metric in laboratory settings. The main focus of the usability assessment was to test the app’s ergonomic design, user-friendliness, comprehensibility, and appropriateness for the target population.

## Methods

### Study Design

The app’s usability was evaluated through a usability test requiring participants to complete a sequence of tasks. The study was performed in 2 stages. After the completion of the first usability test by 10 participants, modifications were implemented in the app based on their feedback. Subsequently, a second usability test, featuring a new cohort of 10 new patients with the same protocol was conducted.

### Participants and Setting

The study was conducted from May 6, 2022, to December 21, 2022, in a multipurpose room at the HUG to standardize the intervention. The evaluation was done through user-investigator interaction, deliberately omitting the user’s real environments. Tasks were performed on an iPhone 6s smartphone (Apple Inc) at a resolution of 1334×750 pixels. For each of the 2 stages, we recruited 2 groups of 10 participants. Ten participants enabled to increase the number of comments and results in accordance with the recommendations which propose at least 5 participants for one arm of a study [26]. Participants were recruited on a voluntary basis from the HUG HF outpatient clinic. Participants had to be adults (aged ≥18 years), speak French, have and use a smartphone, and be a local resident. All enrolled participants had a diagnosis of HF.

The study was divided into 2 stages, each comprising 10 participants (resulting in a total of 20 participants), as proposed by the Nielsen Norman Group [27].

### Cardiomeds Mobile App Overview

Cardiomeds is an mHealth app being developed since 2017 by HUG’s cardiology, pharmacy, medical directorate, and IT departments, with the help of computer scientists, a nurse, pharmacists, physicians, and a student [28]. The app has been designed to fulfill the needs of patients with HF, allowing them to gather and record pertinent data concerning their condition, including treatment regimens, vital sign measurements, and the progression of their symptoms. In addition, the app facilitates data sharing with health care providers and enhances patients’ understanding of their condition through informative quizzes and simplified content. The app also includes the option of setting reminders or alarms to ensure regular and accurate treatment, as well as monitoring patients’ daily medication compliance.

**Figure 1:**
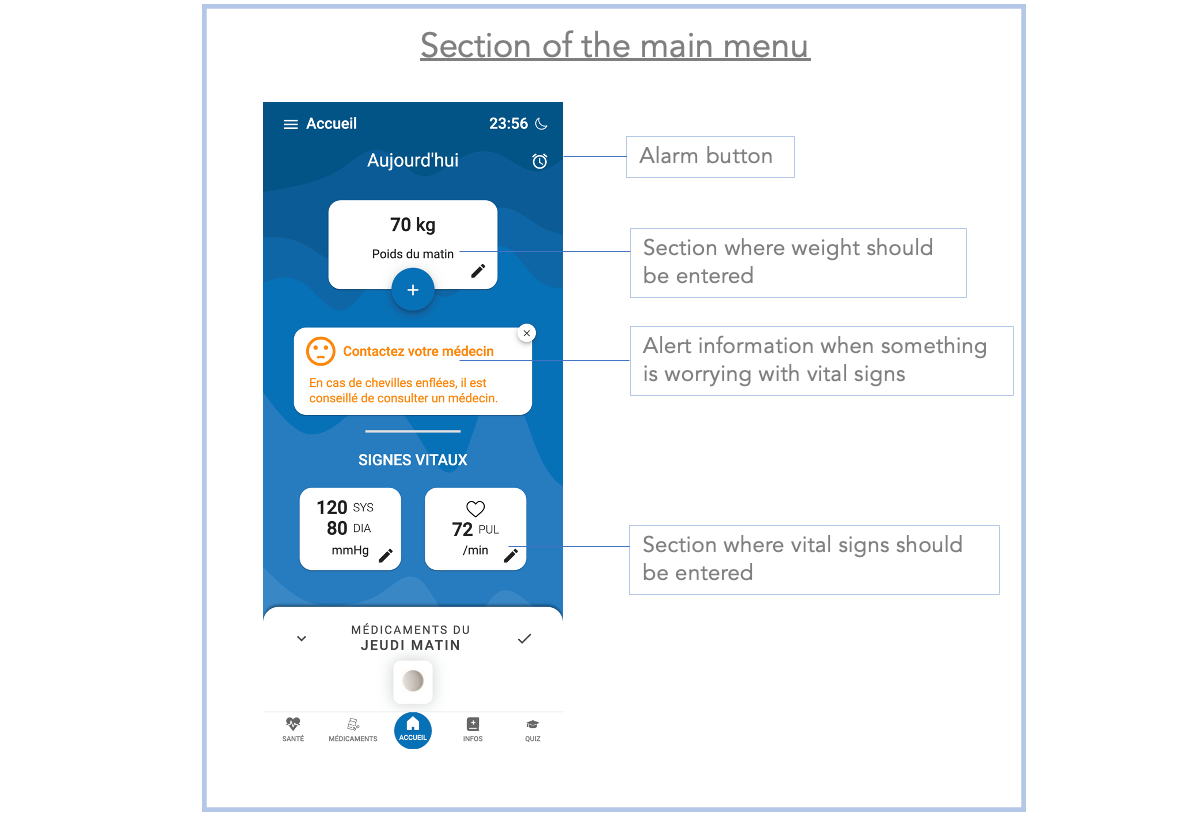
Screenshot of the main menu section of the Cardiomeds app. In this section, patients enter their vital signs of the day, program reminders, and validate their medication intake. Moreover, if their vital signs are not in a healthy range, the app suggests contacting their physician.

**Figure 2:**
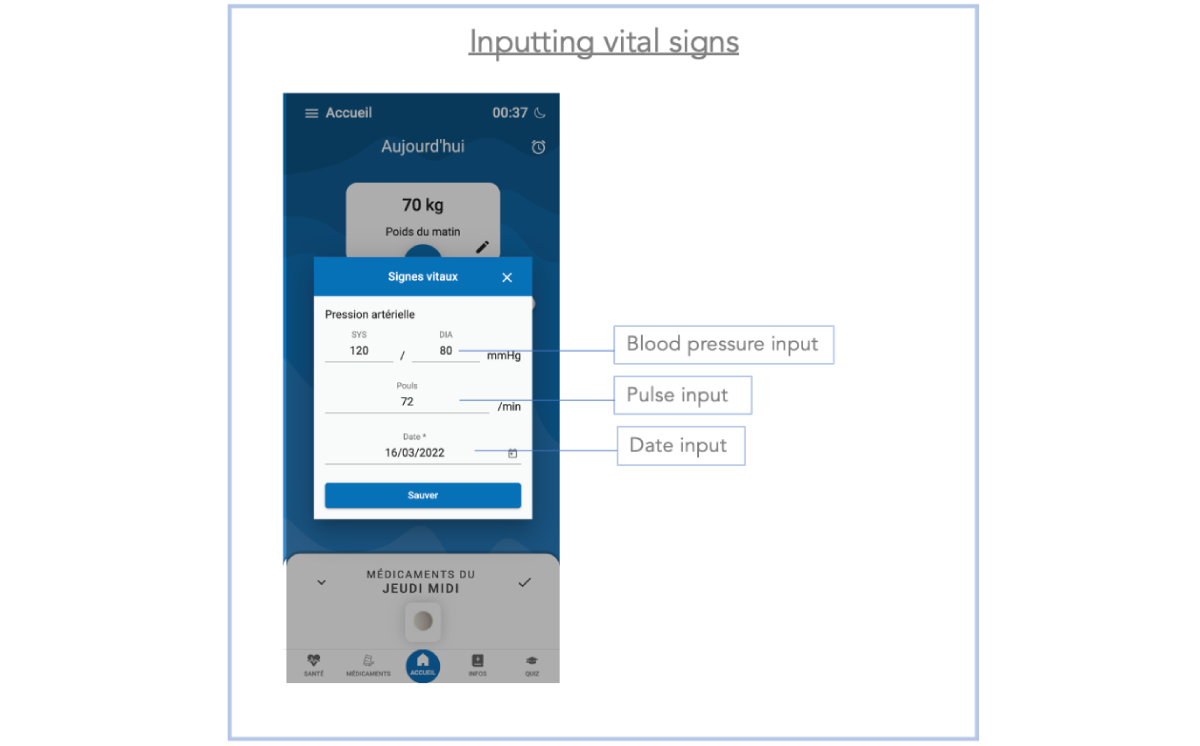
Screenshot of the section where vital signs are entered in the Cardiomeds app. Patients have to enter their systolic and diastolic blood pressure, their pulse, and the day's date.

**Figure 3:**
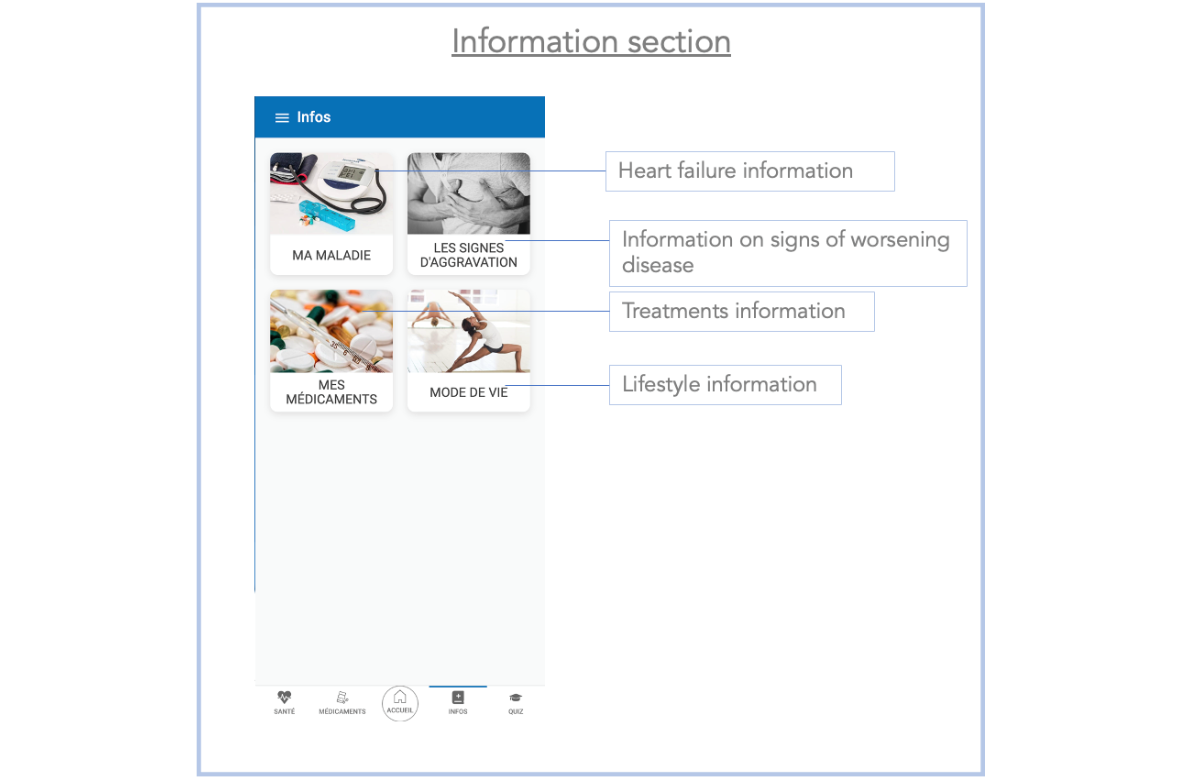
Screenshot of the information section of the Cardiomeds app. Here, patients can find information about their illness, signs of worsening heart failure, their medications, and recommended lifestyle.

**Figure 4:**
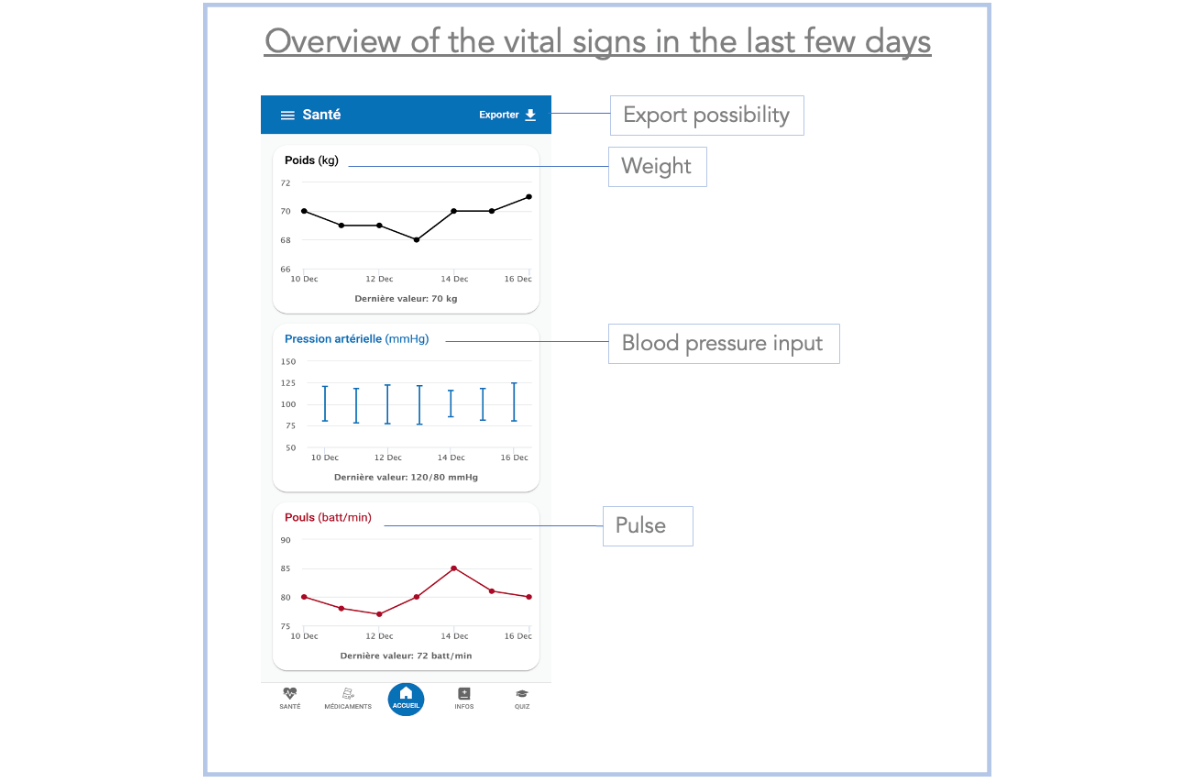
Screenshot of the disease course curve section of the Cardiomeds app. In this section, patients can see the course over the past few days for weight, blood pressure, and pulse. A button to export the curves is also present.

**Figure 5:**
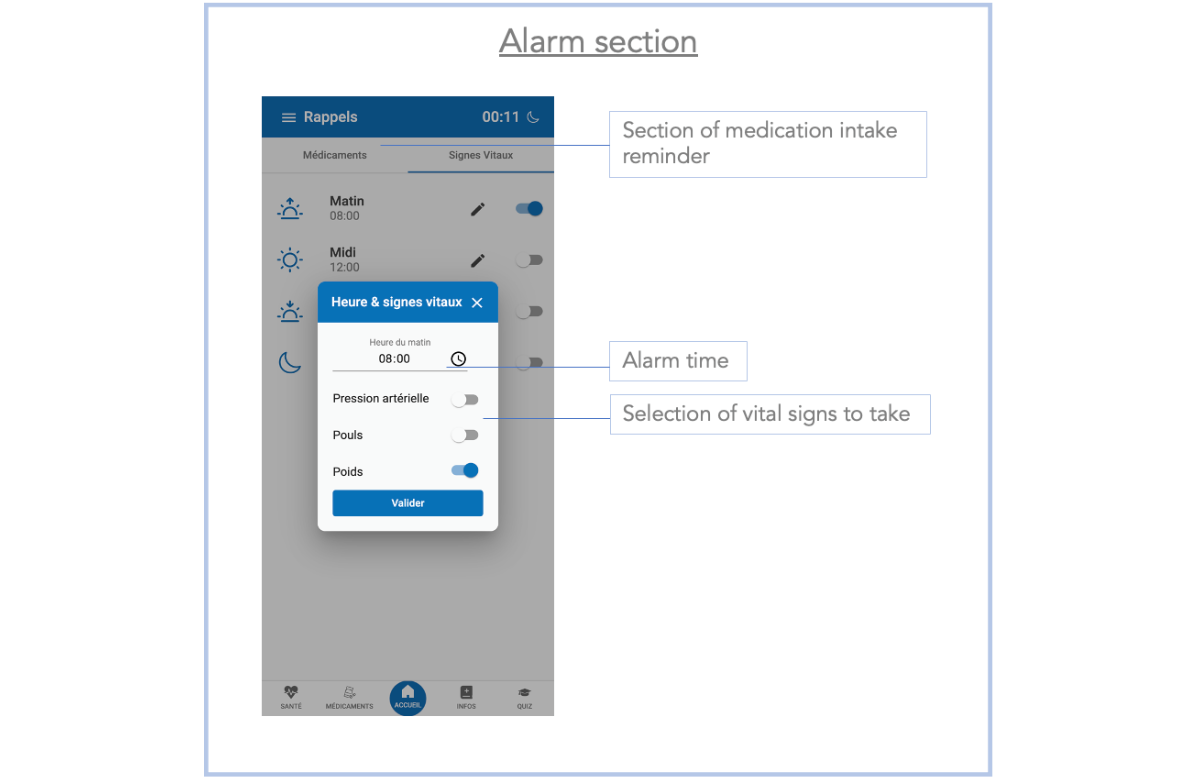
Screenshot of the alarm reminder section of the Cardiomeds app. This section allows the user to set an alarm for taking medication or measuring their vital signs.

The app's ultimate objective is to empower patients by fostering a comprehensive understanding of their medical condition, promoting effective self-management of daily treatments, facilitating continuous clinical monitoring, and enhancing patients’ knowledge of their condition. Figures 1-5 provide an overview of the main menu and the app’s appearance as tested by patients.

### Procedure

All participants were recruited from the HF outpatient clinic of the HUG. They were all invited to join the study via a phone call by a student (LS). Moreover, patients who were selected, were under hospital monitoring, with some having just completed the cardiac rehabilitation program following cardiac decompensation. All interested patients were invited on a given date to take the test. Participants were welcomed within a dedicated room at the HUG, where 2 assigned investigators, a student (LS) and a medical professional (ET), were responsible for overseeing the test procedure. Upon arrival and initial briefing by the investigators, participants signed a consent form, which explicitly stipulated the anonymization of their data.

All participants were provided with a loaned iPhone for test purposes. Then, the test was executed according to the protocol (Textbox 1). Collected measures included task completion timing, recording the activities performed on the smartphone screen, and capturing both the participants’ vocal expressions and the investigator’s commentary. The test comprised 14 predefined tasks exploring the functionalities of the app (Textbox 2). The investigator read out these tasks and precisely timed their execution. Task outcomes were categorized as follows:

- “Passed” if completed within a maximum of 2 minutes without requiring assistance.
- “Partially successful” if completed in more than 2 minutes or necessitating clues provided by the investigator.
- “Failed” if the task was abandoned or remained unsuccessful after 5 minutes.

Upon test completion, each participant was invited to complete the System Usability Scale (SUS) questionnaire [29,30], as well as 5 open questions about their personal feelings about the app and possible improvements (Textbox 3).

Subsequently, data collected from the tests were analyzed using Microsoft Excel. For in-depth insights into the procedures governing each test, please refer to the comprehensive procedure (Textbox 1), as well as the questionnaires distributed to participants (Textbox 3).

**Procedure of the usability test.**

- Welcoming of the user of the app by the investigator.
- Arrival of the user and the worker in a booth.
- Verification of the user’s identity and consent by the investigator.
- Asking for the user’s patient ID, age, gender, education level, smart phone use time per day, and health app use (yes or no)
- Description of the booth:
  - The booth will contain a desk as well as 2 chairs side by side where the user and the investigator can sit.
  - On the desk, 2 cell phones (a Samsung [Samsung Electronics Co, Ltd] phone and an iPhone) will be placed. Therefore, the user will be able to choose the phone whose operating mode they know and with which they are most comfortable.
  - In addition, a sheet on which the tasks to be carried out during the session will be placed on the desk for the user to guide them.
  - Medication packages will also be placed on the desk to carry out the session’s tasks.
- Explanation of the session to the user by the investigator:
  - The investigator will inform the user that their screen and their voice will be recorded (consent).
  - The investigator will explain to the user that the latter will have to carry out the requested tasks, describe their actions out loud, and inform the investigator of the start and end of their tasks.
  - The investigator will explain to the user that after 2 minutes, he or she will gradually give them clues specific to each task to help the user be able to finish the task. If after 5 minutes, the user is unable to complete the task despite help, the task will be marked as failed by the investigator. If the task is completed, the worker will mark it as successful. Once a task is completed, the user can move on to the next task.
  - The investigator will inform the user that he or she can interrupt the session at any time for any reason.
- Start of session:
  - At the start of the session the investigator will set up a screenshot as well as an audio recording if the user gives their consent.
  - The investigator will read the tasks aloud one after the other to the user.
  - Before starting to perform the tasks, the user will have 2 minutes to familiarize themselves with the design of the app.
  - At the beginning, the only questions the investigator will answer will be questions of understanding, if there is a misunderstanding of terminology.
  - The user can reread the task on the sheet placed on the desk for them if necessary.
  - As soon as the user starts each task, he or she will mention it out loud.
  - As soon as the user finishes each task, he or she will mention it out loud.
  - If the user has comments, the investigator will listen to them.
- Tasks: read aloud 14 tasks for the user. Please refer to the tasks below in Textbox 2.

**Description of the 14 goal-oriented test tasks to be performed by users during the usability study.**

- Task 1: set alarm for vital signs (set a reminder to measure your vital signs at 8 AM).
- Task 2: decision-making (go to the home page and enter your morning weight of 65.0 kg). You must then answer 3 questions as follows:
  - Short of breath? As usual.
  - Swollen ankles? More than usual.
  - Any other symptoms? None of the above.
- Task 3: enter weight (you forgot to record your weight on 04.01.2022, and you now want to enter this value, which was 62.3 kg).
- Task 4: enter vital signs (enter your daily vital signs: blood pressure 120/80 mm Hg and pulse rate 70/min).
- Task 5: enter medication (add the medication to the app’s medication list using the available package. Scan the barcode for Beloc ZOK 25 mg. The prescription for Beloc ZOK 25 mg is 2 1⁄2 tablets (2.5 tablets) in the morning every day. In addition, add the number of tablets found in the package).
- Task 6: enter medication without medication package (add a second medication to the app’s medication list. You do not have the medication box. The prescription is as follows: Fosamax [bisphosphonate] 70 mg, 1 tablet on Sunday mornings only; 1 per week. The quantity in the pack is 4 tablets).
- Task 7: enter pro re nata (PRN; taken as needed without a set schedule) medication, without medication package (add a third medication to the app’s medication list. You do not have the medication package. The prescription is as follows: zolpidem (any type proposed) 10 mg, maximum 1 tablet per day, in the evening if necessary. The number of tablets in the pack is 10).
- Task 8: set alarm for medication intake (set a reminder to take your medication at 8:00 AM).
- Task 9: confirm your medication intake (confirm that you have taken your morning medication).
- Task 10: check medication history (check your medication history).
- Task 11: find medication information (you no longer know why you are taking Beloc ZOK [β-blocker]. Where can you find this information? There are 2 ways to find this information).
- Task 12: export vital signs (export your health data [weight, blood pressure, and pulse] to your doctor by email).
- Task 13: quiz (test your knowledge on heart failure with the quiz).
- Task 14: find alcohol information (find information on the possibility of consuming alcohol in the context of your disease).

**Five open questions questionnaire given to users after passing the test.**

1. What is your overall impression of the Cardiomeds app?
2. Do you plan to use Cardiomeds when the app is available, and why?
3. What improvements could we add to the Cardiomeds app?
4. Do you have any other comments about the Cardiomeds app or the usability test?
5. What differences have you noticed between Cardiomeds and other health apps?

### Data Collection

Participants were audio-recorded during the test, and the screen of the test iPhone was recorded. The recording allowed us to retrospectively classify the usability problems encountered with the Bastian and Scapin criteria [31]. The audio was recorded using an iPad-mini-Dictaphone by a single investigator. Moreover, during the test, the investigator timed the tasks with another smartphone. The screen was recorded using the screen recording option built in the iPhone on which the participants were taking the tests. Subsequently, the usability metrics were transcribed onto Microsoft Excel spreadsheets. The audio records were analyzed by the investigator using thematic analysis. The SUS paper questionnaires were collected immediately after the intervention and transcribed onto Microsoft Excel spreadsheets. The investigator analyzed the success rates of each task and their duration. All of the collected data were anonymized. All data described above were collected and analyzed by a single investigator for the 20 participants. ChatGPT (GPT-4 version, OpenAI) was used as an assistance tool to generate text drafts, as a translation tool, and to synthesize concepts. The results were subsequently reviewed and edited by the authors to ensure accuracy and relevance to the topic.

### Usability Analysis

#### Quantitative Evaluation

The participant’s task-based performance was measured by several metrics. Effectiveness is defined by the number of tasks completed by each participant accurately [32]. In this study, effectiveness was calculated in 2 ways: “task completion rate” (TCR) per participant is the percentage of tasks successfully completed [33]. This was calculated using the following equation: TCR per participant=(number of tasks completed successfully/total number of tasks undertaken)×100. (equation 1) When a task could not be started and evaluated (ie, because of a problem with the Wi-Fi connection), it was coded as “unavailable.” TCR per participant was categorized according to 3 different levels of achievement as follows: (1) the task is considered completed when the user has successfully completed the task without any errors or difficulties; (2) completed with difficulty, but with difficulties that could have been solved with the help of the instructor in 5 minutes or less; and (3) failed to complete when the task was left incomplete, abandoned, or the participant took too much time to complete the task (>5 min) [33].

“Distribution of task success by task” was defined as the proportion of participants completing a task according to 3 possible levels of achievement: (1) the task is considered completed when the user has successfully completed the task without any errors or difficulties; (2) completed with difficulty, but with difficulties that could have been solved with the help of the instructor in 5 minutes or less; and (3) failed to complete and the task is left incomplete, abandoned, or the participant took too much time to complete the task (>5 min). When a task could not be started and evaluated (ie, because of a problem with the Wi-Fi connection), it was coded as unavailable [33].

“Efficiency” was defined as the time required for users to achieve specified goals in relation to accuracy and completeness [32]. In this study it was calculated as follows: “time on task” was defined as the average amount of time taken (in seconds) to complete a given task from the moment the participant finished hearing the instructions until the task was completed (whether with ease or with difficulty) or abandoned. When a task could not be started and evaluated (ie, because of a problem with the Wi-Fi connection), it was coded as unavailable.

Satisfaction was measured through the SUS questionnaire. After the completion of the usability test, the SUS questionnaire [29,30] was submitted to each participant. The SUS is a validated questionnaire designed to measure the usability of diverse products and services, including mHealth apps. This questionnaire, conceived by Brooke [29], comprises 10 questions, each offering 5 possible answers, ranging from “strongly disagree” to “strongly agree.” In this ordinal scale, “strongly disagree” corresponds to a numerical value of 1, while “strongly agree” corresponds to a numerical value of 5. The even-numbered questions within the SUS are subjected to negative rotation, and their scores are calculated by subtracting the chosen value from 5. In contrast, the odd-numbered questions undergo positive rotation, and their scores are derived by subtracting 1 from their value. Once the individual scores for all 10 questions were calculated, they are added together and multiplied by a factor of 2.5. Consequently, the resultant SUS score falls within the range of 0 to 100. Usability is considered favorable when the SUS score attains a threshold of ≥70, signifying good usability for the tested product.

#### Qualitative Evaluation

Qualitative data were recorded through an open questionnaire. After completing the SUS questionnaire, participants were asked to answer a series of 5 open questions. The purpose of these questions, defined by the investigators, was to explore the participants’ overall impression allowing us to better understand the strengths and weaknesses of our tools. The 5 open questions are described in Textbox 3.

The responses to the first question were classified into positive responses, negative responses, and no responses. The responses to the second question were classified into 4 categories: yes, no, maybe, and do not know. Questions 3 and 4 were analyzed using the frequency of propositions. All answers to the questions were recorded, sorted, and analyzed by a single person.

#### Bastien and Scapin Classification

We also used the criteria published by Bastien and Scapin [31] in 1993 to evaluate the problems of the app. These criteria cover 8 dimensions and enable the classification of problems between users and the interface.

### Ethical Considerations

On April 1, 2022, a formal request was submitted to the Ethics Commission of the Canton of Geneva to conduct usability tests (Req-2022-00423). This request was subsequently approved on April 4, 2022. The app encompassed the testing protocol, a consent form to be executed by the study participants, and an evaluation sheet for each participant.

## Results

### Participant Characteristics

Between May and December 2022, a total of 20 participants participated in the study. Baseline demographic characteristics of the 2 study stages are shown in Table 1.

Demographic characteristics of study participants (N=10).

**Table 1:** Characteristics of the participants separated into the 2 stages of the study (N=20).

			Stage 1, n (%)		Stage 2, n (%)		Total, n (%)
**Gender**							
	Women	3 (30)		2 (20)		5 (25)	
	Men	7 (70)		8 (80)		15 (75)	
**Age categories (years)**							
	31-40	1 (10)		0 (0)		1 (5)	
	41-50	3 (30)		1 (10)		4 (20)	
	51-60	4 (40)		5 (50)		9 (45)	
	61-70	0 (0)		4 (40)		4 (20)	
	71-80	2 (20)		0 (0)		2 (10)	
**Type of phone**							
	iPhone	3 (30)		1 (10)		16 (80)	
	Samsung	7 (70)		9 (90)		4 (20)	
**Educational level**							
	PRE^a^ graduate school	5 (50)		5 (50)		10 (50)	
	Graduate school	2 (20)		1 (10)		3 (15)	
	University	3 (30)		4 (40)		7 (35)	
**Use of other medical apps**							
	Yes	4 (40)		2 (20)		6 (30)	
	No	6 (60)		8 (80)		14 (70)	
**Time of phone use (h)**							
	<1	1 (10)		0 (0)		1 (5)	
	1-3	8 (80)		7 (70)		15 (75)	
	>3	1 (10)		3 (30)		4 (20)	

^a^PRE: federal diploma of vocational education and training.

### First Stage of the Study

#### Quantitative Evaluation

##### Effectiveness per Participant

**Figure 6:**
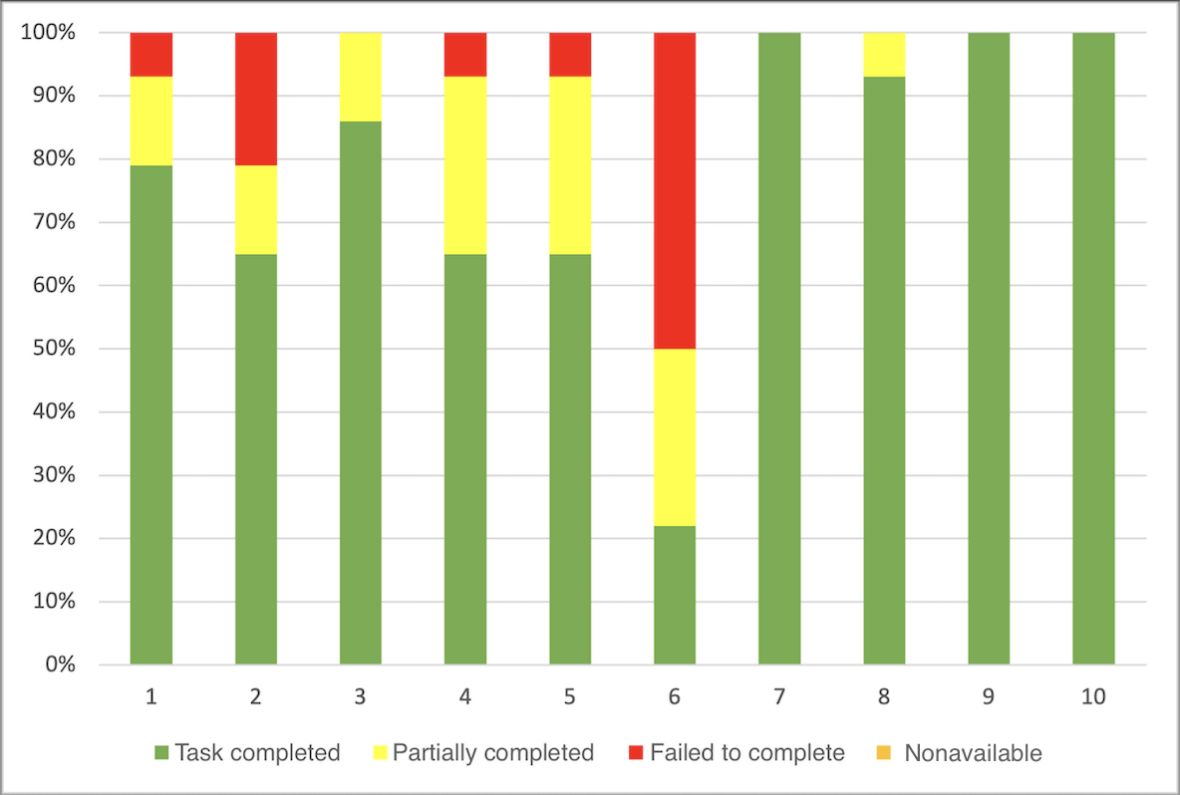
Task completion rate per participant for the 14 assigned tasks during the first stage of the study. "Task completed" represents the percentage of tasks successfully completed by a participant. "Failed to complete" is the percentage of tasks that participants failed to complete. "Partially completed" is defined as tasks completed, but with difficulty that could have been solved with the help of the instructor in 5 minutes or less. "Nonavailable" represents the percentage of missing data when a task could not be started and evaluated.

In the first part of the study, the overall completion rate (tasks completed, partially completed, and failed to complete) was 100% (140/140; Figure 6). The mean overall complete success rate was 50% (5/10), meaning that 50% of the participants solved all the tasks without making any mistakes (they did not fail to complete any task) and 30% (3/10) of the participants completed all the tasks without any help. Participant number 6 failed 50% (7/14) of the tasks and completed only 20% (3/14) of them without any help. Finally, on average, participants completed 77% (11/14; SD 0.23%) of tasks without any help. All these measurements are exposed and supported in Figure 6.

##### Task Success Distribution per Task

**Figure 7:**
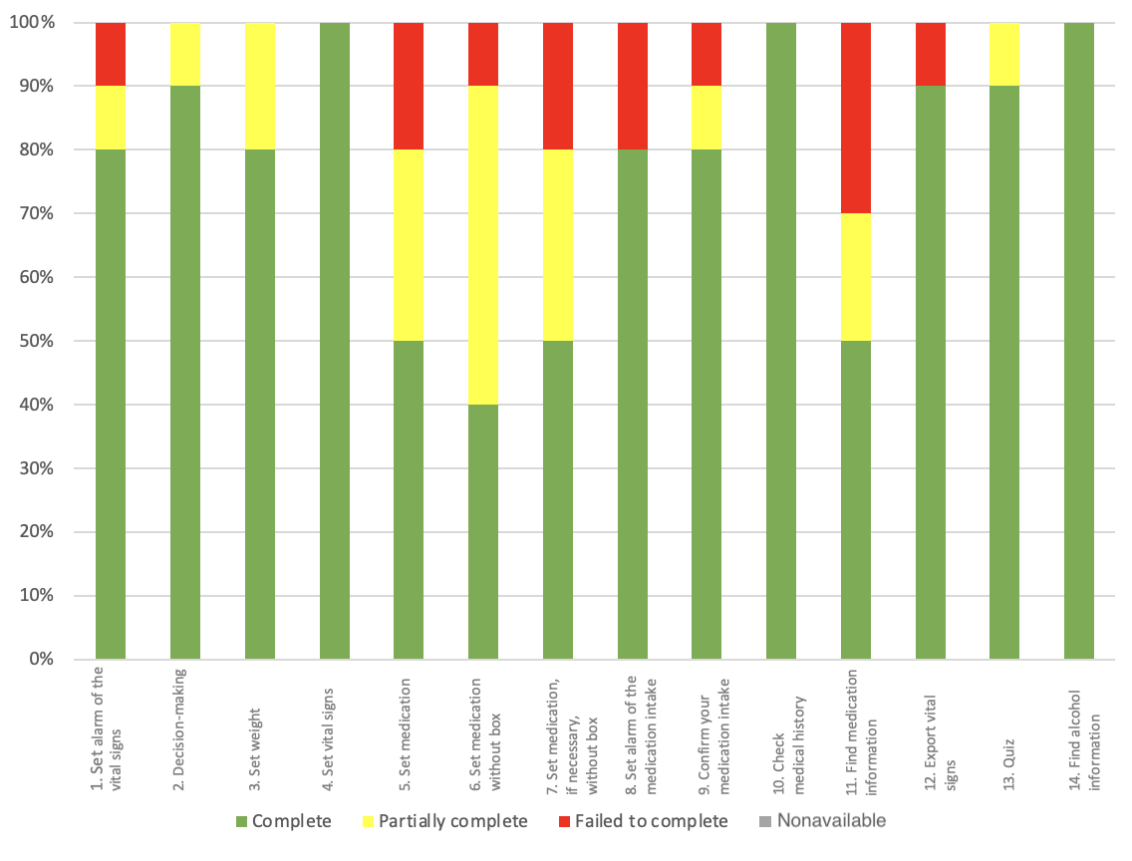
The success distribution per task (N=10 participants) for the first stage of the study. "Complete" represents the percentage of tasks successfully completed by a participant. "Failed to complete" is the percentage of tasks that participants failed to complete. "Partially complete" is defined as tasks completed, but with difficulty that could have been solved with the help of the instructor in 5 minutes or less. "Nonavailable" represents the percentage of missing data when a task could not be started and evaluated.

In the first part of the study, overall 77.1% (108/140) of tasks were completed successfully, 13.6% (19/140) were completed partially, and 9.3% (13/140) were not completed. A total of 21% (3/14) of the tasks (4, 10, and 14) were completed by all the participants without any help (10/10, 100%), followed by task 2 (9/10, 90%). Apart from tasks 4, 10, and 14, 79% (11/14) of the tasks led to difficulties for up to 60% (6/10) of the participants (task 6). At least 1 (1/10) participant failed to complete 8 (8/14) of the tasks (1, 5, 6, 7, 8, 9, 11, and 12), with up to 30% (3/10) of the participants failing to complete task 11, which consisted of finding information about medications in 2 different ways. All these measurements are exposed and supported in Figure 7.

##### Time on Task per Study Task

**Figure 8:**
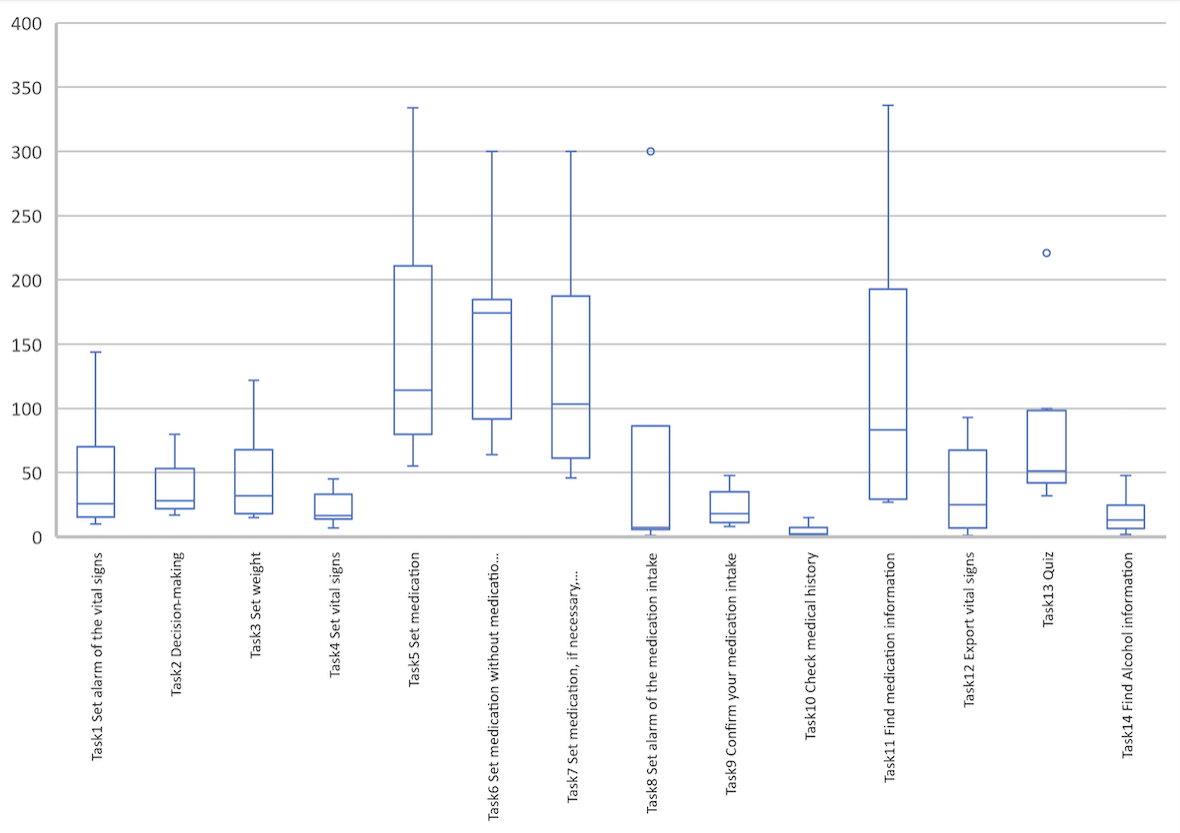
Boxplot of the time taken (in seconds) to perform each task in stage 1.

In the first stage of the study, task 6, which required the participants to enter medication, took the longest time with an average of 154.40 (SD 68.08) seconds (Figure 8). The fastest task completed was checking medication history (task 10) with an average of 4.8 (SD 4.60) seconds.

#### Qualitative Evaluation

##### Five Open Questions Questionnaire

In stage 1, 80% (8/10) of the participants answered positively, 10% (1/10) answered negatively, and 10% (1/10) gave no answer about their overall impression. A total of 70% (7/10) of the participants said that they would like to use the app in the future, 20% (2/10) answered they would not, and 10% (1/10) did not answer the question. The participants who did not want to use the app mentioned that they already had an mHealth app or found that the app was too complicated for them. Participants suggested 3 different types of improvements for the app as follows: first, to increase the font size of the app; second, to change the medication input; and third, to improve the explanation given about the HF drugs. These comments are more described in Textbox 4.

**List of comments made by participants (N=10) that are useful in the short- and long-term for improving the app during the first stage of the test.**

**Useful comments for short-term improvements**

1. The 2 (20%) oldest participants mentioned that the font in the app was too small to understand, which may be problematic for older users.
2. One (10%) participant was interested in transferring his data, but also in having contact with his cardiologist directly via the app.
3. Six (60%) participants found that entering the information on medications was complicated.
4. One (10%) participant wished to have the emergency number, the number of his physician, his cardiologist, and his pharmacy inside the app.

**Useful comments for long-term improvements**

1. One (10%) participant was interested in a search function in the information part.
2. One (10%) participant wished to have interactive blood pressure and saturation connected with his smartwatch.
3. One (10%) participant wanted this app to be connected with his other apps linked to the University Hospital of Geneva Swissmeds app.

##### Error Classification With the Bastien and Scapin Criteria

In stage 1, we recorded 22 problems during the completion of the 14 tasks. We have classified the problems into 9 categories according to the criteria of Bastien and Scapin [31] (Table 2). Task 6, requiring entering a medication, was the most problematic, with 6 (N=10, 60%) users experiencing problems. The screen enabling manual medication recording was considered too dense with information.

**Table 2:** List of problems encountered by participants (N=10) during the 14 tasks and description, categorization, and frequency of occurrence of these problems during task completion.

Item	Category (number according to the Bastien and Scapin classification)	Description of the problem	Task in which it occurs (frequency of occurrence)
1	1. Guidance	Five (50%) users did not find the place of the button to put a reminder. For most of them, it was too small, and its location was not easy to find.	Set alarm for the vital signs (5)
2	1. Guidance	Three (30%) users said the “information” symbol was too small next to the treatments list and that they did not find it easily	Find medication information (3)
3	7. Significance of codes	Two (20%) users did not find the scanning possibility option in the app explicit enough	Enter medication (2)
4	2.2 Information density	Six (60%) participants had difficulties recording medication manually in the app. They say they had to put too much information on one page.	Enter medication without medication package (6)
5	7. Significance of codes	One (10%) participant did not understand names of some drugs because the name of the medication was not written next to the active substance	Find medication information (1)
6	1.3 Immediate feedback	One (10%) participant found the validation of medication intake not explicit enough	Confirm your medication intake (1)
7	7. Significance of codes	Two (20%) participants did not understand the term “if necessary,” such as medication to take in reserve	Enter pro re nata, if necessary, without medication package (2)
8	8. Compatibility	One (10%) participant asked to set an end date for his treatment, but this function does not exist	Enter medication (1)
9	5.2 Error message quality	When entering the date, one participant (10%) did not write it correctly (separated by “/” symbols and not “.” symbols). Therefore, the date could not be validated and no explanation appeared	Enter weight (1)

##### Satisfaction: SUS Questionnaire

**Figure 9:**
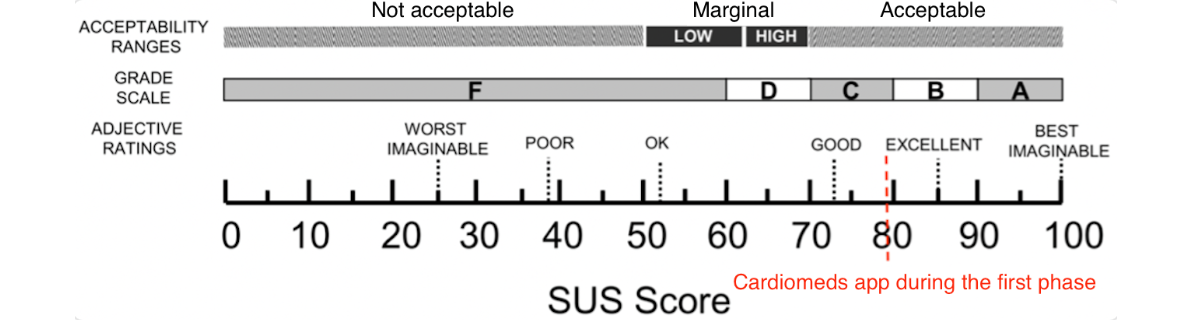
Cardiomeds score on the System Usability Scale (SUS) at stage 1.

At the end of each test, all participants were asked to complete the SUS questionnaire [29,30]. The mean SUS score of the first stage of the study was 79.28 (SD 16.35), (Figure 9; Table 3).

**Table 3:** System Usability Scale (SUS) questionnaire responses in the first stage of the study.

	Question 1	Question 2	Question 3	Question 4	Question 5	Question 6	Question 7	Question 8	Question 9	Question 10	SUS score (sum×2.5; maximum 100)
Participant 1	4	4	3	4	2	2	3	4	3	3	80
Participant 2	3	3	3	3	3	4	3	4	3	1	75
Participant 3	4	4	4	4	4	4	4	4	4	4	100
Participant 4	4	1	2	1	4	3	3	3	3	2	65
Participant 5	4	4	4	3	3	4	3	4	4	4	92.5
Participant 6	4	1	0	0	4	4	4	0	3	0	50
Participant 7	4	4	4	4	4	4	0	4	4	4	90
Participant 8	4	4	4	4	4	4	4	4	4	2	95
Participant 9	2	3	3	4	3	3	3	2	4	4	77.5
Participant 10	2	4	4	4	4	4	3	4	4	4	92.5
Mean (SD)	3.5 (0.82)	3.2 (1.19)	3.1 (1.22)	3.1 (1.40)	3.5 (0.81)	3.6 (0.82)	3 (1.1)	3.3 (1.29)	3.6 (0.52)	2.8 (1.40)	79.28 (16.35)
Median (IQR)	4 (4-3.25)	4 (4-3)	3.5 (4-3)	4 (4-3)	4 (4-3)	4 (4-3.25)	3 (3.75-3)	4 (4-3.25)	4 (4-3)	3.5 (4-2)	80 (92.5-70)

### Improvements Made Between the 2 Stages of the Study

**Figure 10:**
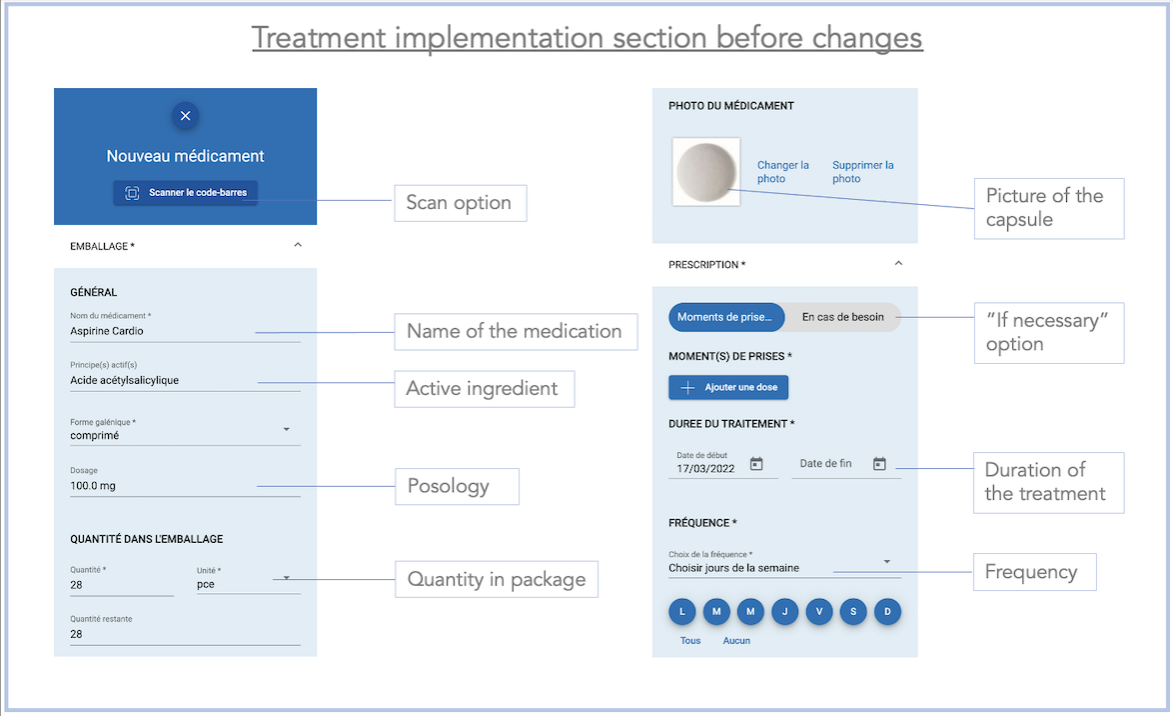
Screenshot of the rolling menu for new medication, before improvements were made, during stage 1 of the study. All the process was presented on one page. First, the patient had to insert the name of their medication and then the time of the intake. There was also a scan button to scan the barcode of the medication.

**Figure 11:**
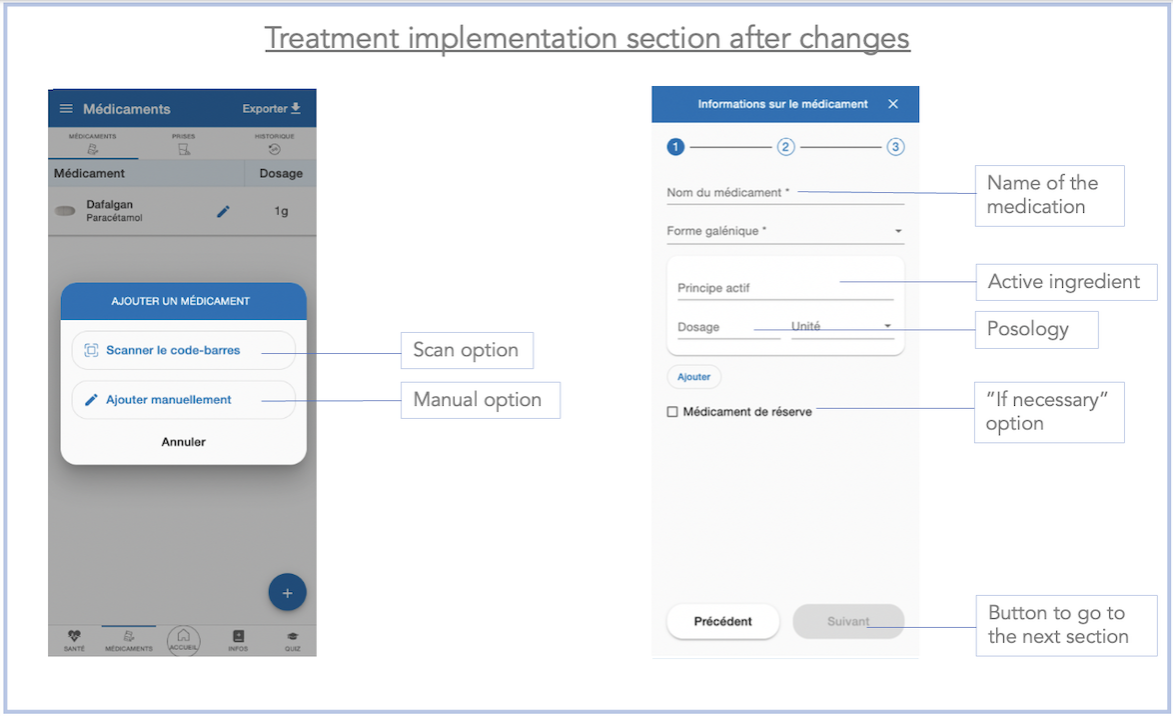
Screenshot of the medication intake section of the Cardiomeds app after the improvements were made during stage 2. First, the patient had to enter the name of the medication or scan the barcode. Then, they pressed the “next” button to go to the next section.

**Figure 12:**
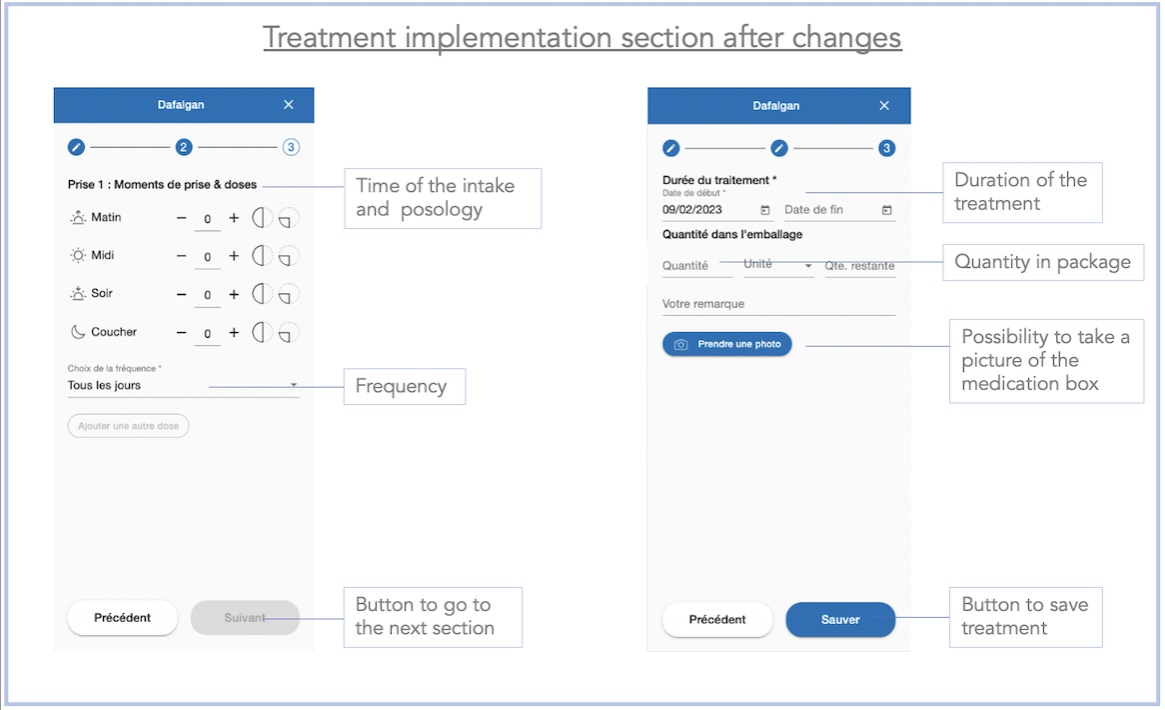
Screenshot of the medication section of the Cardiomeds app after implementation of the changes during phase 2. The patient reached this section after entering the name of the medication or after scanning the medication barcode. Here, the patient had to select the time to take the medication, the dosage, and the frequency.

After completing the test with the first 10 users, we realized that many users encountered difficulties entering medication without scanning the package (task 6). In addition, the users provided comments suggesting the need to facilitate the recording of medications. During stage 1, the manual recording of medications was carried out in a single page (Figure 9). To simplify this process, the ergonomist proposed splitting the process into 3 pages, each requiring a validation (Figures 10-12). We also made some minor changes, such as modifying the localization and visibility of the reminder and information sections to make them more accessible and more visible from the main menu. Once the modifications were done, we began stage 2 of the study. However, we did not increase the font size in the app, despite the many comments we received as mentioned earlier. This did not seem necessary because the font on the smartphone can be changed in its own settings, allowing each user to adapt it to their desires and needs.

### Second Stage of the Study

#### Quantitative Evaluation

##### Effectiveness per Participant

**Figure 13:**
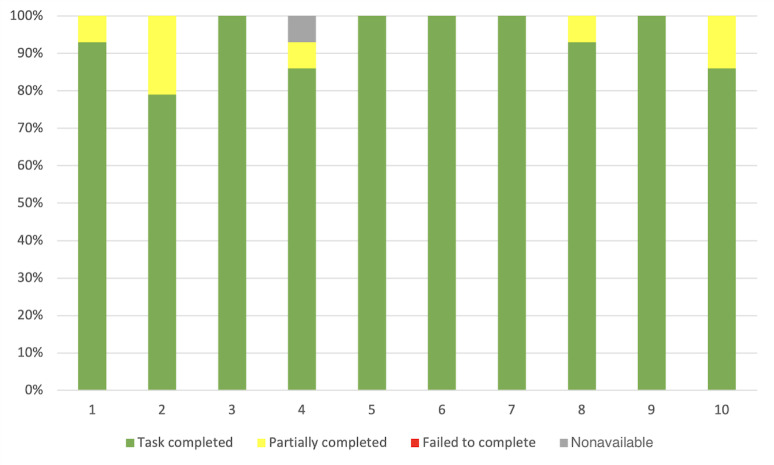
Task completion rate per participant for the 14 assigned tasks during the second stage of the study. "Task completed" represents the percentage of tasks successfully completed by a participant. "Failed to complete" is the percentage of tasks that participants failed to complete. "Partially completed" is defined as tasks completed, but with difficulty that could have been solved with the help of the instructor in 5 minutes or less. "Nonavailable" represents the percentage of missing data when a task could not be started and evaluated.

In stage 2, the overall completion rate of the test (tasks completed, partially completed, and failed to be completed) was 99.3% (139/140). Participant 4 did not perform task 14 during his test because of a software bug. None of the participants failed to complete any task. Only 50% (5/10) of the participants needed help to complete at least one task. Participant 2 needed the most help to finish the test, with 20% (3/14) of the tasks completed with help. All these measurements are exposed and supported in Figure 13.

##### Task Success Distribution per Task

**Figure 14:**
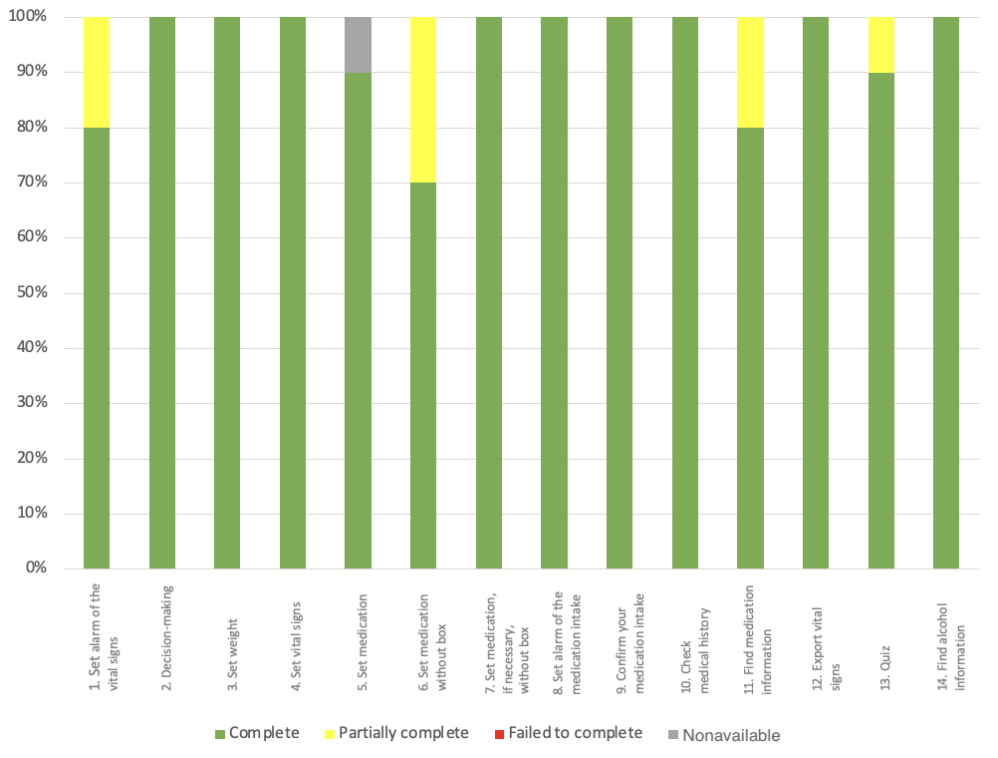
Task success distribution per task (N=10 participants) for the second stage of the study. "Complete" represents the percentage of participants who completed the task with ease. "Partially complete" represents the percentage of participants who completed the task with difficulty that could have been solved with the help of the instructor in 5 minutes or less. "Failed to complete" is defined as the percentage of participants who failed to complete the task. "Nonavailable" represents the percentage of missing data when a task could not be started and evaluated.

In stage 2, overall 93.6% (131/140) of tasks were completed successfully, 5.7% (8/140) were completed partially, and 0% (0/140) failed. A total of 64% (9/14) of tasks 2, 3, 4, 7, 8, 9, 10, 12, and 14 were completed successfully by all participants (100%, 10/10), followed by task 13 (9/10, 90%). A total of 29% (4/14) of the tasks 1, 6, 11, and 13 were completed with difficulty with a rate ranging from 70% to 90%. In this stage of the study, no task completion error was made. Task 5 (enter medication) is the only one with 10% (1/10) missing data because of a technical problem during the test. During this task, the photo scan of the phone did not work. All these measurements are exposed and supported in Figure 14.

##### Time on Task per Study Task

**Figure 15:**
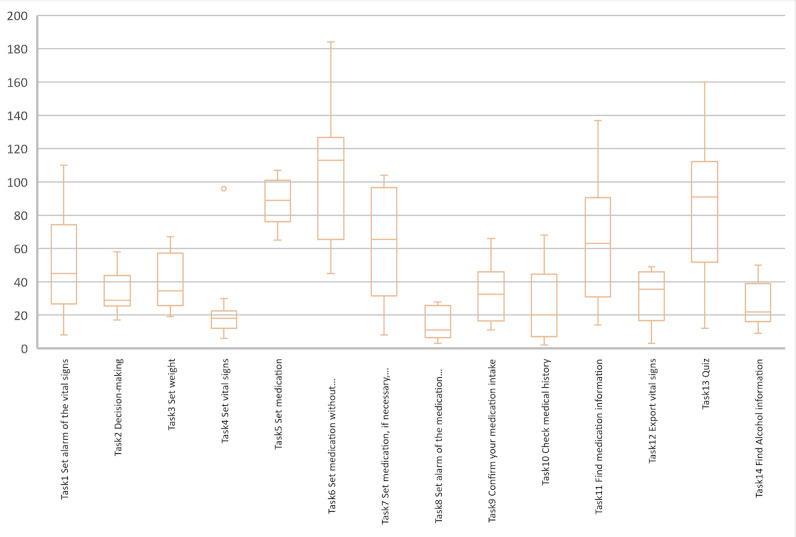
Boxplot of the time taken (in seconds) to perform each task in stage 2.

In the second part of the study, entering medication details (task 6) remained the longest in duration with an average of 103.10 (SD 42.76) seconds (Figure 15). The task that was completed the fastest was task 8 with an average of 21.10 (SD 25.69) seconds.

#### Qualitative Evaluation

##### Five Open Questions Questionnaire

In stage 2, 80% (8/10) of participants answered that their impression of the app was “positive,” 0% answered negatively, and 20% (2/10) did not answer this question.

Furthermore, 100% (10/10) of the participants answered that they would like to use the app.

During the second part of the study, participants suggested making the validation of medication intake more explicit, adding some emergency numbers inside the app, and putting the name of the drug next to the name of the active ingredient of the drug to increase patients’ understanding of their treatments (Textbox 5).

**List of comments made by participants (N=10) that are useful in the short- and long-term for improving the app during the second stage of the test.**

**Useful comments for short-term improvements**

- One (10%) participant suggested making the validation of drug intake more explicit
- One (10%) participant wished to have the emergency number and the number of his physician, his cardiologist, and his pharmacy inside the app

**Useful comments for long-term improvements**

- One (10%) participant wanted to have the name of the medication next to the active principle in the medication information

##### Error Classification With the Bastien and Scapin Criteria

In the second part of the study, we recorded a total of 15 problems. We classified them into 7 categories according to Bastien and Scapin’s criteria [31] (Table 4). The most problematic were tasks 9 (confirm your medication intake) and 12 (export vital signs), with 4 (N=10, 40%) users experiencing problems with each task. Participants reported that the validation of daily medication intake was not explicit enough. The participants who encountered a problem with task 12 found that the “export” button was too small and therefore not legible and that the location of this button was not well placed.

**Table 4:** List of problems encountered by participants (N=10) of the second part of the study during the 14 tasks and description, categorization, and frequency of occurrence of these problems during task completion.

Item and problem	Category (number according to the Bastien and Scapin classification)	Description of the problem	Task in which it occurs (frequency of occurrence)
1	1. Guidance	Five (50%) users found the front text size too small and the localization of some symbols not easy to find	Enter medication (1) and export vital sign (4)
2	1.3 Immediate feedback	Four (40%) participants did not easily understand how to validate a medication intake	Confirm your medication intake (4)
3	7. Significance of codes	One (10%) participant did not understand some names of drugs because the name of the medication was not written next to the active substance	Find medication information (1)
4	1. Guidance	One (10%) user did not find the medication information in the principal menu	Find medication information (1)
5	5.2 Error message quality	When entering the date, two (20%) participants did not write it correctly (separated by “/” symbols and not “.” symbols). Therefore, the date could not be validated, and no explanation appeared	Enter weight (2)
6	5.2 Error message quality	One (10%) participant made a mistake while entering his vital signs and could not go back, so he had to restart the task from the beginning	Enter vital signs (1)
7	2.2 Concision	One (10%) participant was troubled by the fact that there are two places to find information on drugs	Find medication information (1)

##### Satisfaction: SUS Questionnaire

**Figure 16:**
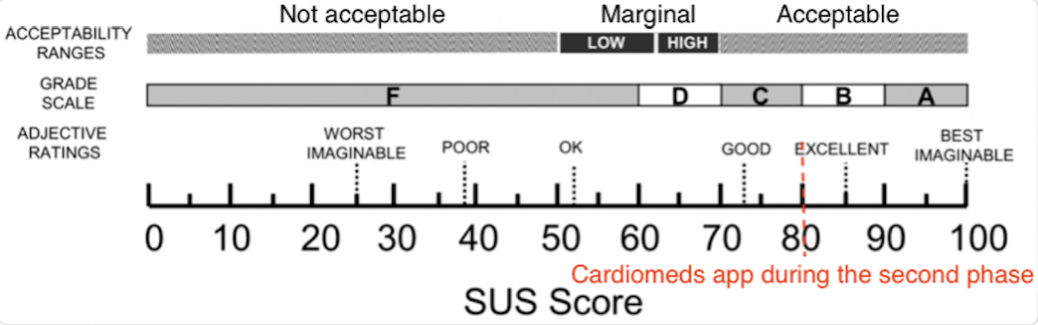
System Usability Scale (SUS) score on the SUS [35] of the Cardiomeds app at stage 2.

The mean SUS score of the second stage of the study was 80 (SD 28.3), described in Table 5 and classified in Figure 16.

**Table 5:** Responses to the System Usability Scale (SUS) questionnaire in the second stage of the study.

	Question 1	Question 2	Question 3	Question 4	Question 5	Question 6	Question 7	Question 8	Question 9	Question 10	SUS score (sum×2.5; maximum 100)
Participant 1	3	3	3	4	3	3	3	3	3	3	77.5
Participant 2	3	0	3	3	3	4	2	3	3	0	60
Participant 3	3	4	4	4	4	4	4	4	4	4	97.5
Participant 4	3	4	3	4	4	4	4	4	4	3	92.5
Participant 5	4	4	4	4	4	4	4	4	4	4	100
Participant 6	4	3	3	3	3	3	3	3	3	3	77.5
Participant 7	3	4	4	4	3	4	3	4	4	4	92.5
Participant 8	3	4	3	4	3	4	3	4	3	4	87.5
Participant 9	4	3	3	4	3	4	3	4	4	1	82.5
Participant 10	3	1	1	1	3	3	3	2	3	1	52.5
Mean (SD)	3.3 (0.47)	3 (1.34)	3.1 (0.83)	3.5 (0.93)	3.3 (0.46)	3.7 (0.5)	3.2 (0.6)	3.5 (0.68)	3.5 (0.52)	2.7 (1.52)	80 (28.3)
Median (IQR)	3 (3.75-3)	3.5 (4-3)	3 (3.75-3)	4 (4-3.25)	3 (3.75-3)	4 (4-3.25)	3 (3.75-3)	4 (4-3)	3 .5 (4-3)	3 (4-1.5)	80 (90-70)

### Comparison of First and Second Stages

#### Quantitative Evaluation

##### Task Success

**Figure 17:**
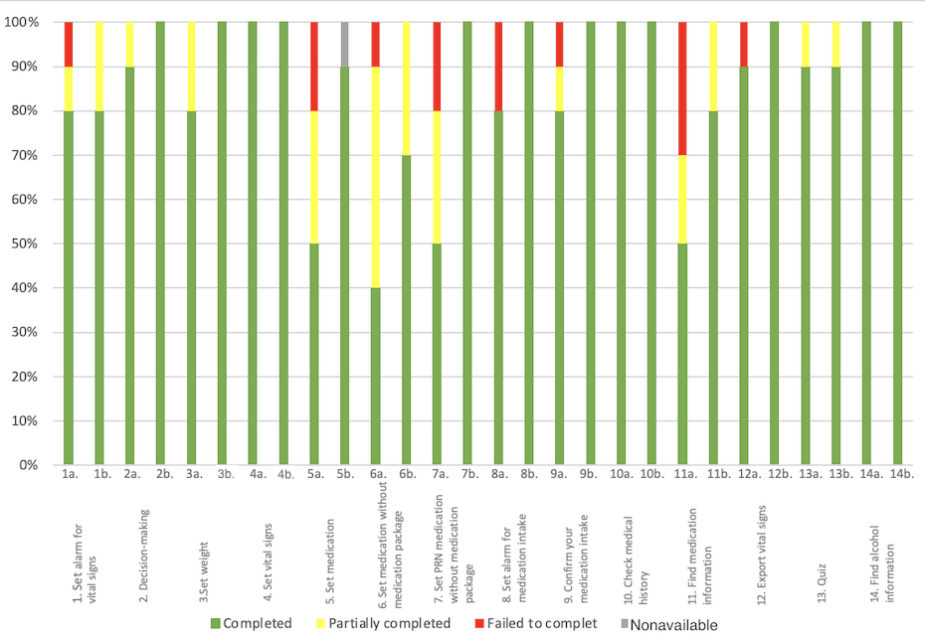
Task success distribution per task (N=20 participants) for the overall study. "Completed" represents the percentage of participants who completed the task with ease. "Partially completed" represents the percentage of participants who completed the task with difficulty that could have been solved with the help of the instructor in 5 minutes or less. "Failed to complete" is defined as the percentage of participants who failed to complete the task. "Nonavailable" represents the percentage of missing data when a task could not be started and evaluated.

On average, participants completed 86% (12/14; SD 0.19%) of tasks without any help overall. Conversely, on average, participants needed help with 14% (SD 0.11%) of tasks. As depicted in Figure 15, tasks 4, 10, and 14 maintained a success rate of 100% (20/20) in both stages of the study. Tasks 2, 3, 7, 8, 9, and 12 had a successful completion rate without any help of 100% in the second stage of the study. We also noticed that the failure rate for all tasks combined was reduced to 0% in the second stage of the study. All these measurements are exposed and supported in Figure 17.

##### Time on Task per Study Task

**Figure 18:**
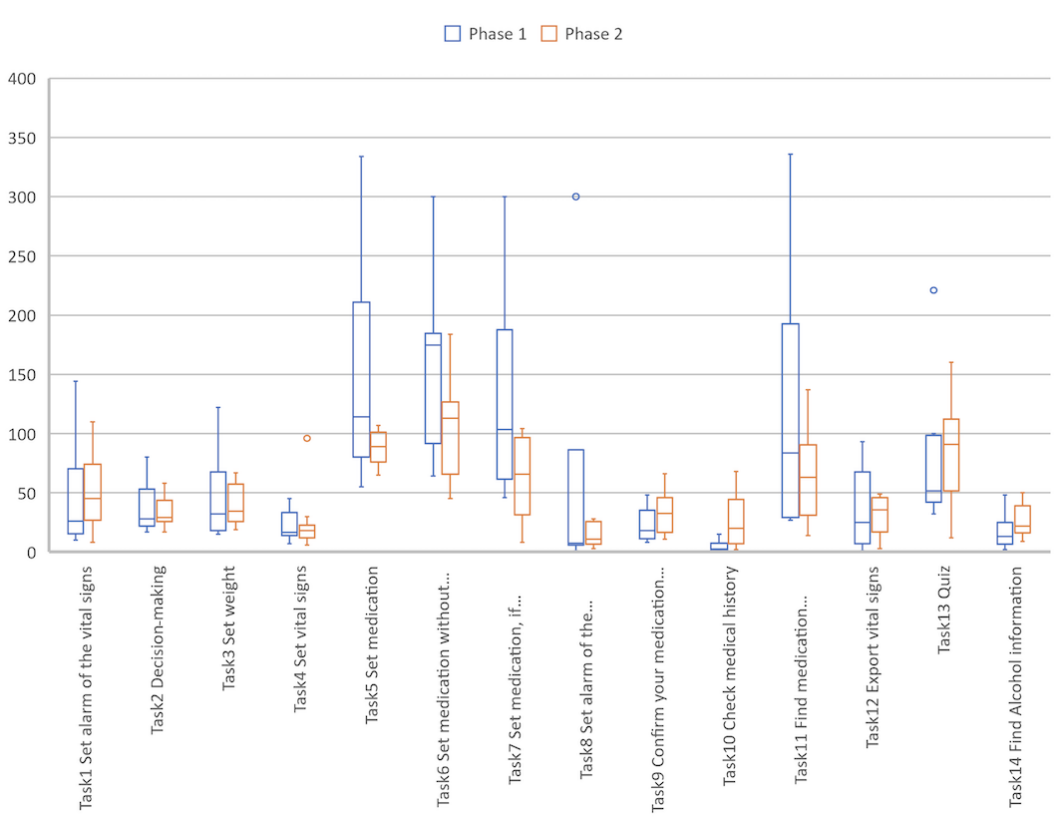
Boxplot of the comparison of the efficiency of the 2 stages of the study. Stage 1 is shown in blue and stage 2 is shown in orange.

The mean overall time on task for all tasks was 57.19 (SD 40.05) seconds. Tasks 5, 6, and 7 had a longer duration than the other tasks in both stages of the study (Figure 18). Nevertheless, task 11 was also one of the longest during the first stage. These findings showed that the task with the most failures (ie, task 6 [enter medication without medication package]) was also the most time-consuming in both stages of the study, although the time taken was reduced during stage 2.

#### Qualitative Evaluation

##### Five Open Questions Questionnaire

Regarding the qualitative part of the study, 80% (16/20) of participants responded positively to our open questions about general feelings in both stages of the study. Between the 2 stages, the number of participants who had negative feelings was reduced from 1 participant to 0 participant. In addition, 85% (17/20) of participants were inclined to use the app once it became available, indicating its ease of use. Another comment that emerged in both stages of the study was the wish to access various contact numbers (4/20, 20%), such as the emergency, physician, cardiologist, or pharmacy.

##### Error Classification

The first stage of evaluation highlighted various ergonomic problems as defined by the Bastien and Scapin criteria [31]. The reduction, from 22 problems of 6 different types in the first stage of the study to 15 problems of 5 different types identified during the second stage, demonstrated the benefits of the improvements made to the app.

We observed that in both stages of the study, the most frequent problem remained the problem of guidance (first stage; 8/22, 36%, and second stage; 6/15, 40%).

It seems that the change in localization of the reminder and information section led to a reduction of guidance problems, although 2 different types of problems remained, including 1 new problem (Table 6). Given that guidance problems appeared several times in both stages of the study for tasks 8, 11, and 12, we could improve the app further by making the buttons of the reminder and the information section even more visible in a future version (reminder, export vital signs, and find medication information). A recommendation would be to add a label under the reminder button and increase its size. As for the button in the information section, we would like to change its color and increase its size.

**Table 6:** Type of problems during stage 1, number of occurrences, number of new problems, and total type of problems in stage 2.

Category (number according to the Bastien and Scapin [31] classification)	Stage 1	Stage 2		
	Types of problems	Solved problems	New problems	Types of problems
1. Guidance	2	1	1	2
5.2 Error message quality	1	0	1	2
7. Significance of codes	3	2	0	1
8. Compatibility	1	1	0	0
2.2 Information density	1	1	0	0
1.3 Immediate feedback	1	0	0	1
2.2 Concision	0	0	1	1

Moreover, we observed that the problem of the significance of codes decreased from 22% (5/22) to 7% (1/15) and from 3 such problems to only 1 (Table 6). These problems appeared during the task in which the participants had to scan the medication package, which we have made easier and more visible within the app, but also about the appellation by the component of certain medications that we have also tried to popularize as much as possible to reach a wider audience, for example, we have inserted the names of the drugs (metozerok) next to the class of the drug (β-blocker).

Problems related to error message quality (from 1/22, 5% to 3/15, 20%) and immediate feedback (from 1/22, 5% to 4/15, 26%) increased, although there was no new problem of the immediate feedback type, unlike the error message quality for which a new type of problem appeared. These problems did not increase significantly and are strongly dependent on the participant samples. Finally, a new problem of concision was mentioned during stage 2 by a single participant concerning the location of information concerning medications which could be found in 2 different places.

##### Satisfaction: SUS Questionnaire

**Figure 19:**
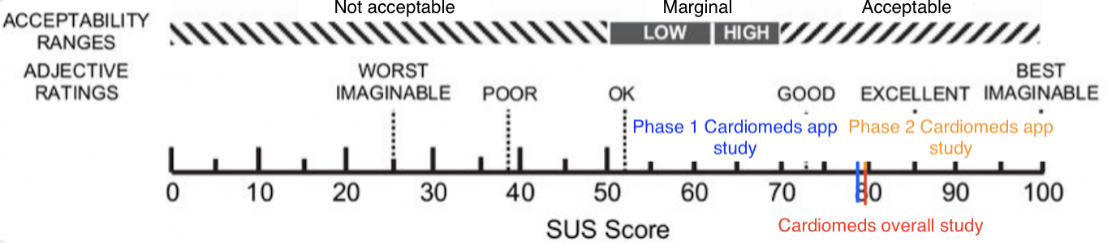
Cardiomeds score on the System Usability Scale (SUS) for the overall study.

Averaging the 2 results of the SUS questionnaire gave a score of 80 for the entire study. This shows that the usability of the Cardiomeds app was perceived as good in both stages of the study with a slight improvement during the second stage after some modifications. The scale in Figure 19 is used to interpret these results [34].

## Discussion

### Principal Findings

Participants took 18 minutes on average to complete all 14 tasks, which means just over 1 minute per task. However, certain tasks took much longer than others. The shortest task took an average of 4.80 (SD 4.60) seconds to be completed in the first stage (task 10) and 21.10 (SD 25.69) seconds in the second stage (task 8). One possible reason that tasks 8 and 10 were the shortest in the study could be that these 2 tasks required minimal navigation within the app. In addition, for task 8 (set alarm for medication intake), participants had already set an alarm for task 1 (set an alarm for vital signs), which likely made this task easier. Similarly, task 10 (check medication history) followed task 9 (confirm your medication intake), where the same menu also provided access to the medication intake history button. Finally, because both tasks followed task 6, which was the longest in the study, participants may have become more familiar with the app’s navigation, helping them complete the later tasks more efficiently. Furthermore, between the 2 stages of the study, we modified the localization and visibility of the reminder icon to make it more accessible and more visible from the main menu. This might explain why task 8 (set alarm for the medication intake) was the fastest task. However, we were unable to determine why the duration of task 10 (check medical history) was longer in the second part of the study than the first because we did not make any change that could influence retrieval time.

The maximum average time was 154.40 (SD 68.08) seconds during the first stage (task 6) and 103.10 (SD 42.76) seconds during the second stage (task 6). The entry of medications (task 6) took the longest time in both stages of the study, although we observed an improvement between the first and the second stages, suggesting that the app had been improved. Improving the medication entry process was crucial to reduce the time patients spent inputting or modifying their treatment in the app. We know that patients with HF with reduced left ventricular ejection fraction usually take at least 4 different medications. In addition, the older the patients, the more they suffer from different comorbidities, such as type 2 diabetes [35] or hypertension [36], which further increases the number of medications required. Furthermore, the treatment for patients with HF is likely to change often, so we wanted to make this part as fluid, ludic, and easy to use as possible. Knowing these considerations, the time spent on the app could significantly increase which could discourage the target audience from using it. Therefore, it was important to improve the ergonomics of drug recording to make this process as short as possible. Feedback collected on task 6 (enter medication without medication package) led to app modifications aimed at simplifying the medication entry process. The main change was to divide the drug recording process into 3 different pages rather than having a single scrollable page. Nevertheless, we believe that after repeated use, the time taken will diminish, and the patient will become increasingly comfortable to use the app. Also, although entering medications is an important initial process in using the app, it is not needed in daily use.

Concerning the quantitative results of the study, we observed that the mean overall success rate, which represents the effectiveness (completed the task without any help and completed the task with help) was 77.1% (108/140) for the first stage and 93.6% (131/140) for the second stage of the study. Furthermore, only 30% (3/10) of the participants in the first stage of the study managed to complete all tasks without any help versus 50% (5/10) during the second stage. We wonder if the differences observed between the 2 stages are partly due to the difference in characteristics of the participants in the 2 groups. Stage 1 included more participants aged between 71 and 80 years (1/10, 10%) than stage 2 (0/10, 0%).

We observed that 8 tasks (8/14, 57%) were failed by at least one participant during stage 1 compared to none during the second stage. The tasks failed or completed with difficulty in the first stage of the study included tasks 5 (5/10, 50%), 6 (6/10, 60%), and 7 (5/10, 50%). All these tasks were related to the recording of the medication. These same tasks induced fewer problems during the second stage of the study, with only 30% (3/10) of the participants completing task 6 with difficulty, and none of the participants making any errors for tasks 5 and 7. As these tasks aimed at the recording of medications, we can reasonably assume that the changes made to the app after the first stage contributed to the reduction of problems encountered during the second stage of the study.

Finally, we observe in Figure 19 that the efficiency of task 10 (check medical history) seems worse in the second stage of the study (mean time for the overall participants 27.10, SD 21.70 seconds) than in the first stage (mean time for the overall participants 4.80, SD 4.60 seconds). On the basis of our observations, we suspect that during the first stage of the study, participants took more time to complete the previous tasks, which gave them more time to navigate through the app. Thus, we hypothesize that the medication history page was already localized by the participants in stage 1.

**Figure 20:**
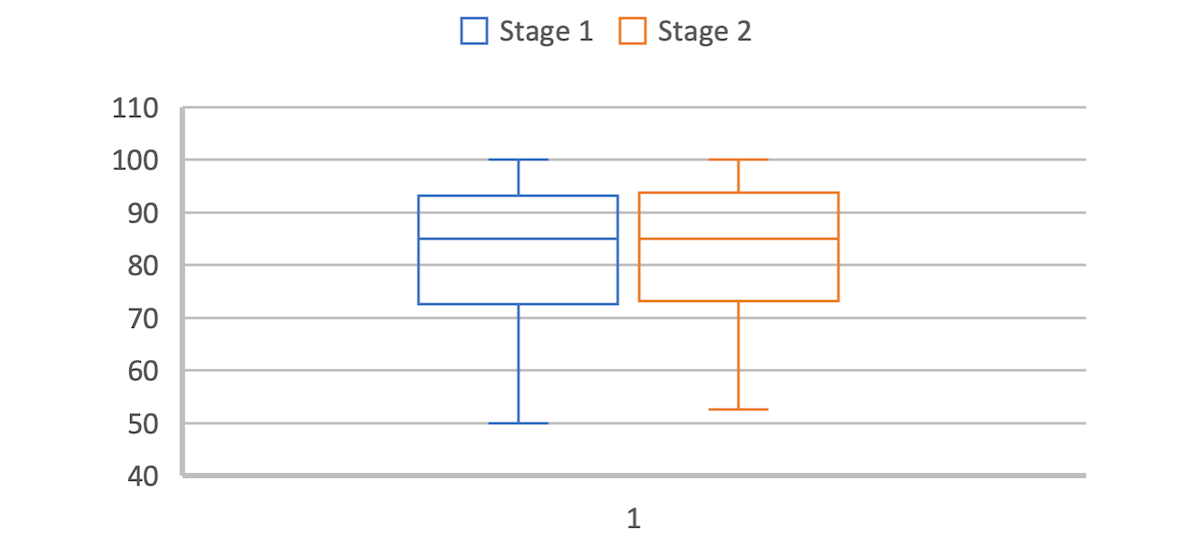
System Usability Scale score distribution in both groups.

It is surprising to observe that the significant increase in effectiveness per participant, from 50% (5/10) in the first stage of the study to 100% (10/10) in the second stage, is not reflected in an increased SUS score. Indeed, the Cardiomeds app was first evaluated with usability measured as “good” with a SUS score of 79% on average. It improved by 1% to reach 80% after modifications were made to the app between the 2 stages of the study. Nevertheless, when we look at the distribution of scores in the 2 groups in Figure 20, we see that they are very similar. We concluded that the participants mainly judged the concept of the app rather than the difficulty of the tasks carried out during the usability tests through the SUS. Therefore, it is logical that despite the changes made to the app, the SUS score did not change much.

During the tests, we collected various participants’ comments. In the first stage of the study, the most common comment (2/10, 20%) was the extremely small font size, which may be a problem for older users. We did not change the font size in the app because it is possible to do so globally in the settings of each device. During the second stage of the study, this remark did not emerge again, although we did not change the font size.

Furthermore, we can observe in Table 6 that between the 2 stages of the study, the problems of information density and compatibility were solved. Indeed, these problems mainly appeared while entering medications without a medication package (task 6), whose interface was improved between the 2 stages of the study. As mentioned earlier, this stage has been improved because we believe that the recording of medications must be simple given the number of treatments necessary and their regular change depending on the clinical condition of the patients and the course of their illness. We decided not to categorize guidance problems because it was not clear whether the guidance problems were localization problems or legibility problems. Indeed, the guidance problems were found in task 8 (set alarm for medication intake), 11 (find medication information), and 12 (export vital signs). It was not clear if the button was too small or poorly located, which would not make it visible enough.

It is important to point out that despite the availability of mHealth health interventions, most patients still wish to have follow-up appointments with their physician to maintain supervision and human contact. Many patients express the fear of being harmed when relying only on a health app [37]. However, with the current shortage of physicians and nurses in Switzerland’s urban areas [38], the management of these patients is complex. The follow-up made possible with an app, such as Cardiomeds, could probably provide relief and help physicians to focus more on the relationship with the patient during medical appointments. Moreover, when patients are involved in their care and carry out self-checks, they are also more involved in their health, thus enabling in-depth monitoring of their illness to best prevent cardiac decompensations and avoid hospitalizations. Therefore, it is clear that physicians need to add eHealth to their daily practice by integrating it into their management, to offer optimal long-term follow-up.

### Strengths

The first strong point of our study was that although common guidelines assume that a sample of 5 users is sufficient to reveal 85% of usability problems and 15 users is sufficient to discover almost 97% of problems [39], we evaluated the usability of the Cardiomeds app with 2 groups of 10 people each. In addition, we split the study into 2 stages, which enabled us to make modifications based on the feedback and then test the app with a new group to see if the changes improved the app’s usability.

Second, we used mixed methods combined with usability testing methods to report and analyze quantitative and qualitative data in this study, which is recognized as a strong point in studies analyzing the usability of apps [40,41].

Finally, in our usability testing protocol, users had to complete tasks like those they would perform alone at home. Studies have shown that this is a good way to test an app’s usability. In addition, the inclusion criteria for our study were broad, allowing a large panel of patients with HF to participate. In addition, even if most participants did not have an iPhone, it was still possible for Android users to test the app.

### Limitations

A few limitations need to be considered. First, we can see that the most represented age group in our study (50-60 years) is younger than the age of the audience targeted by the app. Indeed, HF is more prevalent in patients >65 years of age [42]. Second, in both groups, women were underrepresented, with an average of 25% (5/20) of women, which is less than the prevalence of HF in women, which reaches 50% [42]. Third, only one investigator oversaw data collection in this study. Indeed, the usability tests, the SUS score, and the 5 open questions questionnaire were evaluated by one investigator, and all the recorded data were analyzed and timed by a single investigator. As only one person measured the times, there is a risk of bias in the results; however, this would be minimal given that the same investigator performed all the recordings.

Finally, the SUS questionnaire has limitations in assessing the usability of apps. Indeed, the SUS questionnaire does not provide specific insights into which parts of the app may cause issues for users. Moreover, it is a subjective measure of perceived usability. By adding some open questions to this questionnaire, we were able to obtain more information, but this does not reduce the inherent deficiencies of the SUS [43,44].

### Conclusions

According to the SUS score obtained in both stages of the study, our usability study rates the Cardiomeds app as “good,” with a slight improvement of 1% between the 2 stages of the study, to reach 80% in stage 2. The effectiveness per participant (the number of tasks completed or partially completed by each participant), went from 50% in stage 1% to 100% in stage 2. The most time-consuming task in both stages of the study was manual medication recording (task 6). Nevertheless, the improvement made after the first stage of the study enabled a reduction in the necessary time to complete this task. The most frequent ergonomic problems according to the Bastien and Scapin criteria remained problems of location and legibility within both stages of the study, with a slight improvement in stage 2. The problem of information density was solved because of the improvements made to this app by the IT professionals regarding medication recording. Most participants who tested the app recommended it and said they would use it when available. The multidisciplinary care required by patients with chronic HF is often complex because of the presence of many comorbidities. Therefore, it would be interesting to enable them to better understand their disease and increase their involvement, improve their therapeutic adherence, and finally enhance their quality of life through the future use of this mHealth app.

## Abbreviations

HF: heart failure
HUG: University Hospital of Geneva
mHealth: mobile health
SUS: System Usability Scale
TCR: task completion rate
